# HRM and organizational learning in knowledge economy: investigating the impact of happiness at work (HAW) on organizational learning capability (OLC)

**DOI:** 10.1186/s43093-023-00188-2

**Published:** 2023-03-08

**Authors:** Safwat Adel El-Sharkawy, Muhammad Salah Nafea, Emad El-Din H. Hassan

**Affiliations:** Faculty of Management Sciences, October University for Modern Sciences and Arts (MSA), Cairo, Egypt

**Keywords:** HAW, Work–life balance, OLC, Total reward management, Knowledge management

## Abstract

The purpose of this paper is to examine the main antecedents of happiness at work (HAW) as a main driver of organizational learning capabilities (OLC) among academic staff working in Egyptian private universities. The mediating role of HAW between these drivers like Work–Life Balance (WLB) and Recognition from one side and OLC on the other side has also been evaluated. A survey-based research strategy has been adopted. A survey of 207 academic staff employees working in Egyptian private universities was conducted to test the direct effects of the hypothesized relationships. The findings of this study supported the hypotheses that recognition has significant positive total effect on OLC and partially through the mediating effect of HAW among academic staff at Egyptian private universities. In addition, WLB also was found to have a significant positive total effect on OLC and partially through the mediating effect of HAW. Finally, it was concluded that HAW has a significant positive total effect on OLC and partially mediates the Recognition-OLC and WLB-OLC links. Accordingly, this research provides crucial and major implications for both HR professionals and the top management of Egyptian private universities through considering factors like recognition and WBS while designing an effective total reward system that reinforces the overall organizational learning capabilities in the emerging markets context.

## Introduction

“Our staff are our most important asset.” Many business leaders and managers have intoned this mantra a year after a year, but many of employees have probably thought deeply to themselves that their managers place a higher value on the machines in a factory or even to the cash in a bank account. Some countries’ governments around the globe have turned their nations all-in on positivity and happiness. For instance, the Emirati Government have presented and installed a giant smiley face on the wall in most of Dubai police stations. In addition, they have created and established a Ministry of Happiness and started funding researches in happiness and happiness at workplaces as well.

Not only that, but also, a lot of awards and funds have been devoted toward creating happiness at workplaces. Per example, the Happiness @ Work Award was launched in UAE during the year 2018 in partnership and cooperation with Forbes Middle East. This award was created in parallel with the UAE overall happiness strategy and supported by Sheikh Mohammed bin Rashid Al Maktoum, the Prime Minister of the UAE and Dubai Ruler. In fact, this award was all set to recognize these corporate entities in the region that offers its people the highest degrees of happiness and comfort. The award’s jury evaluates those nominated corporates and picks the happiest workplace across five different categories: The Best Workers’ Welfare Program, Workplace Wellness Program, the Best Work–Life Balance Program, the Best Employee Engagement Program in addition to the Best Workplace Sustainability Program.

Therefore, the main research question addressed in this research is demonstrated as follows: What is the role of recognition and WLB as antecedents of OLC through the mediating effect of HAW? Hence, the purpose of this study is to examine the conceptual and empirical impact of recognition and WLB as main drivers of OLC. In addition, the research aims at developing a framework for assessing the mediating role of HAW as a mechanism through which the perception of total reward benefits components such as Recognition and WLB affect OLC.

## Need and rationale of the study

In today’s highly competitive work context, human resources (HR) are presumed to be a critical and primary source of organizational success and achieved competitive advantage. In particular, multifarious researches in the human resources management (HRM) domain are skewed toward topics of the organizational performance. Accordingly, shedding a light on the employees’ role in today’s dynamic work environment would be a tunneling contribution to three different areas of knowledge in HRM domain: knowledge management, total rewards management and subjective well-being (Happiness).

Moreover, multifarious evidences have suggested that a lot of research works have been conducted on rewards, HAW and their consequences on OLC in the western context [40]. However, the researchers in the current study propose that a little focus on the eastern and Arabian context. In other words, the total rewards management models developed in the western context are not necessarily applicable to the eastern one. In the other words, few studies have highlighted these motivational influences of total rewards mix in different contexts. Employees of different countries have demonstrated preferences for a different mix of reward components for the sake of achieving HAW [43]. For instance, multifarious researches reveled that employees in western contexts feel HAW if they found their work interesting, whereas employees in eastern cultures feel HAW once they feel secure at their jobs [73]. Accordingly, the findings of the current literature postulated that the main components of total rewards management that directly affect employees’ HAW are totally contextual. Therefore, despite the unstoppable flow of these researches investigating the linkage between total rewards management and HAW, few researches have examined this relationship in the eastern context. Consequently, the current research could be an appropriate contribution to eastern and Arabian context of HRM literature.

Furthermore, while investigating the body of knowledge for these studies linking the total rewards management, HAW and OLC, Chiva et al. [20] found that there is a need to investigate the relationship between HAW and OLC as both variables directly affect the overall organizational performance and meanwhile effectiveness. There is a lack of empirical evidence that has investigated this relationship in the eastern context.

## Theoretical framework and hypotheses development

### Happiness at work (HAW)

This study postulates that happiness is the ultimate pursuit of people across the globe. The real definition of happiness differs according to the perspective of an individual person or even a group of people in this dynamic and challenging world. One of the major types intensively investigated in the literature of HRM is Happiness at Work (HAW). Large pile of researchers has indicated that HAW has a considerable influence on employee performance [78], employees’ mental health [94], employee creative performance [56], and psychological capital [58]. Recently, during the prolonged lockdown, the organizations' mitigation efforts to control the spread of the coronavirus were work from home initiatives to enhance happiness at work and overall productivity [65]. While Thompson and Bruk-Lee [95] highlight that high job demands reduce employee happiness, which will increase turnover intentions and counterproductive work behaviors. Additionally, happy workers and employees can get promoted faster, getting more support from their own supervisors, tend to effectively accomplish their own tasks and generate new innovative ideas for their own organizations [43].

In fact, multifarious researchers have thought of the definition of HAW from different perspectives. For instance, Gulyani and Sharma [43] have defined HAW as the extent to which an employee can experience acceptable levels of satisfaction and positive affections at workplaces. Despite of the importance of achieving HAW, many organizations encounter a lot of challenges concerning how to achieve it. Accordingly, many researchers revealed that these positive feelings among workers and employees could be achieved through well-tailored and crafted total rewards programs [10].

Moreover, the theoretical foundation based on which this research is built is mainly derived from the Social Exchange Theory (SET) developed by Blau in 1964. The SET entails that employees tend to be willing to contribute to their organizations in terms of knowledge and skills, only if they feel that their employers are concerned for their welfare or when their organizations grant them adequate and reasonable job resources [43].

In general, past evidence from the body of knowledge has revealed that employees may feel positive regarding their workplace when they receive different types of rewards: material, social and non-monetary rewards [1]. In more details, social rewards are present in terms of recognition and good relations with managers and colleagues, whereas non-monetary rewards such as achieving work–life-balance (WLB) could be factors that contribute to achieving acceptable levels of HAW [1].

### Recognition

Employee recognition has got attention from scholars and practitioners as it is one of the most effective strategies to motivate employees at organizations [2, 14, 15, 34, 49, 60, 62, 67]. In contemporary organizations, employee recognition is one of the most effective techniques to enhance leadership effectiveness, task performance and employee citizenship behavior. Employee recognition is typically a leader-adopted constructive feedback practice based on value assessments of specific employees, such as work performance and dedication, and correlated with positive outcomes [103]. While Lartey [60] highlights that employee recognition has a vital influence on employee engagement which the theoretical model of social exchange endows. From another perspective, Montani et al. [67] concluded that various sources of employee recognition, particularly manager and coworker recognition, could effectively influence behavioral involvement in the workplace.

The concept of employee recognition is mainly focused on non-monetary rewards to emphasize the desired behaviors that employees are required to perform [62]. On the other hand, the literature points out that recognition alone will not be effective unless it is coupled with monetary rewards [28]. Correspondingly, the majority of organizations combine both recognition (non-monetary) and rewards (monetary) to elicit the outstanding performance of their employees [5]. Employee recognition historically reinforces the desired behavior [92], increases citizenship behaviors [32], lower resistance to change [33], ensure the success of planed change [90], positively influences job performance [30], enhances normative commitment [35] and employee engagement [25, 53, 76]. Although employees’ recognition is vital, the majority of employees stated that they receive less recognition particularly from their supervisors [102]. On the contrary, [98] highlight the importance of social exchange between supervisors and employees which enhances advocacy behaviors through their commitment and endeavors. Moreover, organizations that implement work–life practices such as supervisor work–life support enhance employee performance [69].

#### Employee recognition and HAW

Several studies highlight that employee recognition has a substantial impact on happiness at workplace [4, 6, 18, 57, 59]. [52] hypothesize that recognition is one of the facets of motivation that lead to happiness. The study of De Guzman et al. [27] highlights that employee recognition has a positive influence on happiness at workplace, particularly among a group of aging employees. Moreover, Salas-Vallina Alegre and Guerrero [85] found that recognition facilitates happiness at workplace particularly among knowledge-intensive workers.

Likewise, Awada et al. [8] in their study comprise employee recognition as one of the components of happiness at workplace. Similarly, Salazar [87] stated that recognition and reward programs carried out by Colombian and American corporations enhance happiness at workplace, thus enhance productivity. From another perspective, Khan and Abbas [56] indicated that recognition and rewards enhance happiness and thus creativity. Correspondingly, Chantal et al. [18] claim that employee recognition has a vital influence on increasing organizational productivity and happiness. Thus, employees who feel acknowledged are happy more and confident in contributing to the organization's success.

#### Employee recognition and organizational learning capability (OLC)

Employee recognition has historically been one of the strategic roles that human resources management utilizes to enhance organizational learning through the effective implementation of reward and recognition programs and policies [17, 39]. Thus, employee recognition facilitates the process of shaping the required behavior or standards that the organization seeks to pursue. Similarly, the study by Austin and Harkins [7] indicates that employee recognition played a critical role in facilitating organizational learning as a way to cope with the changes in organizational climate and work environment.

Moreover, Nafukho et al. [71] study highlights the vital role of leadership in promoting organizational learning in small-sized business through the effective implementation reward and recognition systems. Whereas the work of Jain and Moreno [50] includes reward and recognition as one of the components of organizational learning capability construct. From another perspective, Escrig et al. [31] stated that organizations that employ effective HR practices such as recognition and altruistic leader behavior enhance their organizational learning capability and lead to a noticeable radical innovation. In light of previous studies, employee recognition was one of the cornerstones that facilitate organizational learning capability as well as happiness at workplace as represented in the work of.

Furthermore, Afshari and Hadian Nasab [2] claim that recognizing talented employees improves organizational learning capability through the mediating role of intellectual capital. Additionally, Sobaih and Hasanein [93] highlight that employee recognition facilitates organizational learning and enhances its learning capabilities. In light of the above-mentioned relations, the researchers are capable of synthesizing the following hypothesis:

##### H_a_

Recognition has significant positive Total Effect on OLC.

##### H_a1_

Recognition has significant direct effect on HAW.

##### H_a2_

Recognition has significant positive direct effect on OLC.

##### H_a3_

Recognition has significant positive indirect effect on OLC via HAW.

### Work–life balance

Over the last two decades, work–life balance has gained significant attention from organizations that drives them to develop policies and practices regarding work–life balance to provide healthy work environment. The notion of work–life balance was originally theorized as Work–family conflict (WFC) and defined as “a form of inter-role conflict in which the role pressures from the work and family domains are mutually incompatible in some respect” [41]. It employs the role theory to describe the conflict and balance that employees encounter in performing various roles between work and family.

Later, the concept of work–life balance refined and clarified as “Satisfaction and good functioning at work and home with a minimum of role conflict” [21]. While the work of Kalliath and Brough [54] viewed Work–life balance as the employee's perception that the work and non-work demands are compatible and endorse career growth that suits the his/her contemporary life priorities. Consequently, [44] define it as the employee’s perception of how his or her diverse life roles are balanced. Similarly, Hobson [47] states that work–life balance is the balance between work and family duties.

Reaching the unparalleled crisis of the coronavirus pandemic (COVID-19) has paved the way to widely adopt work–life balance policies and practices due to the significant physical, mental, and emotional stress that employees encountered during the pandemic [55]. Whereas Thrasher et al. [96] in their study examine the inter-sectional influence of both age and gender on executives' work–life balance, highlight that male and female manager encounter distinguishable a work–life balance due to dissimilar social roles and managerial expectations. Recently, several studies have utilized occupational stress theory to illustrate work and family as sources of stress that consumes the employees’ time, energy, and attention [104]. Thus, balancing work and family demands has a considerable influence on reducing occupational stress, enhancing employee satisfaction and overall well-being. Thus, the majority of scholars employ two theories in their attempt to crystalize work–life balance which is role theory and occupational stress theory.

#### Work–life balance and HAW

Previous studies highlight the influence of work–life balance on happiness at workplace [9, 26], Wan Mohd [3, 70, 80, 99, 101, 105]. The work of Otken and Erben [74] concluded that there is variance between Generation X and Generation Y in their perspectives on work–life balance and happiness. As Generation X work/personal life negatively affects happiness, while it positively affects Generation Y. Consequently, Generation Y places high value on work–life balance than Generation X. While the study by highlight that to enhance happiness at work, management should promote effective work–life balance for male and female in the organizations.

Whereas, the work of Rao et al. [77] comprises work–life balance as one of happiness at work factors. The study concluded that work–life balance is a critical element of happiness at work particularly during the first stage of employment, due to the various roles required from the employee. From another perspective, Dhingra and Dhingra [29] found that gender has a moderating effect on the relation between work–life balance and subjective happiness. The study reveals that female favor fewer work hours to handle family responsibilities than male doctors.

#### Work–life balance and organizational learning capability

The majority of organizations realize that in order to facilitate the organizational learning process, employees need to balance between work and family responsibilities [38, 82, 83, 97]. While Gomes et al. [38] concluded that work–life balance initiatives facilitate organizational learning capability, leading to high-service innovation. Recently, Charoensukmongkol and Puyod [19] claimed that during the COVID-19 pandemic, transformational leadership positively lowers role ambiguity and promotes the work–life balance, thus enhancing organizational learning capability. Additionally, the previous studies prove that work–life balance motivate employees and upsurges their openness to learning [91]. Similarly, [51] claims that there are problems associated with work–life imbalances which negatively affect organizational learning, such as below-performance, stress, employee frustration, and perceived lack of organizational support. Thus, for organizations to foster their learning capability great attention should be given to work–life balances and practices.

##### H_b_

WLB has significant positive Total Effect on OLC.

##### H_b1_

WLB has significant direct effect on HAW.

##### H_b2_

WLB has significant positive direct effect on OLC.

##### H_b3_

WLB has significant positive indirect effect on OLC via HAW.

### Organizational learning capability (OLC)

In fact, the concept of organizational learning capability (OLC) stresses the role played by these facilitating conditions for reinforcing organizational learning or even organizational readiness or tendency to learn [20]. [37] defined OLC as the organizational facilitating factors that stimulate organizational learning process.

Considerable studies emphasize that organizational learning capability has a critical role in enhancing organizational innovation [45], entrepreneurial intellectual skills, and employees’ innovation capabilities [63], talent management and intellectual capital [2] and organizational performance [48, 68].

In 1959, Peter Drucker presented the concept of knowledge-intensive employees. From this point of time forward, the field of HRM has received an increased attention [85]. Therefore, this study postulates that those knowledge-intensive employees are in an inevitable need for a workplace context full of social interactions for the sake of effective communication, collaboration, creativity and brainstorming with their peers. In parallel with the claim of the current study, Salas-Vallina et al. [85] have proposed developing a workplace or context that improves knowledge-intensive workers’ happiness at work (HAW) will in turn keep those employees feel highly motivated. Multifarious researches revealed that it is of top priority for those knowledge-intensive employees to feel happy at work to reach their own potential.

#### Happiness at work (HAW) and organizational learning capability (OLC)

A large pile of researches has postulated the considerable influence of happiness at work and organizational learning capability [11, 61, 79, 81, 86]. Such studies hypothesize that learning is stimulated through employee engagement and satisfaction, taken into account that both of engagement and satisfaction are all major components and ingredients of the HAW construct. Another perspective toward a deeper understanding for such relationship between HAW and OLC revealed that when employees feel free of risk, they could be easily invigorated toward achieving their utmost performance and utilizing their own individual capabilities. In addition, the previously mentioned relationship could be reinforced and advanced in these situations through which employees feel happy at their workplace [82, 83].

One of the unconventional claims in regard with creating OLC asserted that it requires a complete transformation for the workplace and the surrounding context from the current static state to another level of a more dynamic one [82, 83]. Generally, it requires reshaping the internal systems as a whole. Finally, another pile of researchers revealed that creating a learning organization is a function of a series of actions that have a significant impact in creating OLC such as: continuous learning opportunities, promoting dialogue, collaboration, shared learning concepts, shared vision, employees’ empowerment which could be all considered as ingredients of HAW and totally directing the organization toward creating [13, 36, 75, 82, 95]. In light of the above-mentioned relations, the researchers are capable of synthesizing the following hypothesis:

##### H_c_

HAW has significant direct impact on OLC.

## Methodology

### Research design

Normally, research’s objectives and questions play an integral role in determining the type of research design. For any research, a research paradigm is considered as the holistic framework that guides researchers in every single step in their research journey. As per [24], in social sciences, there are three main paradigms: positivism, interpretivism and pragmatism. The positivism paradigm is the most commonly used approach in social sciences researches; as an individual researcher can evaluate the results without involving personal judgements [72]. Accordingly, the philosophical paradigm utilized in such study falls within the positivism paradigm. Moreover, in light of the positivism paradigm, this research adopts a deductive approach to researches, in order to test the research hypotheses and answer the proposed research questions.

Regarding the current research’s strategy, a survey-based research strategy is adopted as it is considered as a reasonable approach for gathering large number of responses from large number of participants who are geographically dispersed [64]. Furthermore, the research method adopted in the current research under the survey-based umbrella was a quantitative method. [23] promoted the adoption of quantitative research method especially among those researchers who adopt positivism paradigm.

### Sampling and data collection approach

The current research adopted a cross-sectional survey strategy the measurement of a proposed theoretical model; as it did not intend to measure change over time. On the other hands, for data collection, the current research referred to a population of academic staff who have spent a period that exceeds 1 year in their current position regardless of what these positions are in Egyptian private Universities. In fact, data collection has been gathered from different private universities. Due to the absence of a well-defined population frame, a non-probability sampling technique was adopted in this research to pick those respondents of the survey [88]. Specifically, the current research started with a convenient followed by a snowball sampling technique.

In light of non-probability sampling techniques, sampling size may be a question. However, [100] promoted that if smaller sample was satisfactory and carefully picked, there would be no need to draw very large samples. Moreover, it was suggested by Campbell [16] to utilize the following formula (50 + 8*x*) for obtaining the minimum possible size of a sample; where *x* is the number of predictors or exogenous variables. Moreover, for the sake clearly defining the optimal sample size for the current research, the researchers used G*Power 3.1.9.4 freeware package to calculate it. The software parameters were stated as follows: alpha (*α*) was set at 0.1, the effect size (*f*^2^) was a small effect (0.05) as stated by Cohen [22], and at number of predictors or exogenous variables equivalent to Three (2 independents and one mediating variable). Accordingly, the software recommended a minimum sample size of 203 respondents.

Accordingly, for the sake of measuring the proposed relationships among constructs in this research, an online self-administered questionnaire was mainly sent to 614 employees working in Egyptian universities. 209 responses were collected, whereas 207 were utilized for the final analysis, which represented a response rate of 34.8 percent. Two responses were excluded due to having a response pattern. 70 percent of the respondents were females, and 30 percent were males. In addition, participants in this study represented age groups varying between less than 30 to more than 60 years old, with well-diversified educational backgrounds and positions in the academic hierarchy. In general, 28% were teaching assistants (TAs), 27.5% were Assistant Lecturers (ALs), 27.5% were lecturers, 9.7% Associate Professors and 5.3% were Full Professors.

In addition, according to Sekaran and Bougie [89] a pre-test of the survey instrument should be conducted for the sake of establishing content validity. Therefore, experts from different universities at different managerial and academic positions were participated in the pre-test process. Hence, feedback from those experts was acquired regarding the clarity and relevance of the postulated constructs’ items. In addition, the survey instrument was further tested for investigating reliability of all constructs.

### Measures

The current research has utilized standard measures for the sake of effectively operationalizing the core constructs depicted in the proposed conceptual model in this research. Reliability and validity values of all the postulated constructs, in addition to the value of Cronbach’s *α* of all constructs exceeded the cut-off point value of 0.7.

This research measures HAW through a higher order construct that considers three independent scales of employee engagement, job satisfaction and organizational commitment. This scale is adopted from the shortened HAW 9-item scale developed by Salas-Vallina and Alegre [84]. Concerning the measurements of OLC, a 5-point Likert scale validated by [20], where 1 represents “Strongly Disagree” and 5 represents “Strongly Agree.” Four items are comprising the OLC construct such as “People are encouraged to interact with the environment: competitors, customers, technological institutes, universities, suppliers etc.”

On the other hand, a 6-item scale measurement developed by Gropel [42] was utilized to measure the WLB. For instance, the WLB items utilized in this research are: “I often visit my friends and acquaintances,” “Because of my work, I have no free time” and “Because of my work, I neglect my family or friends.” These items are assessed by Cronbach α, and an internal consistency was revealed with 0.81 and 0.75.

## Results and findings

### Factor analysis to remove redundancy

The primarily use of factor analysis is to reduce dimensionality of data to a lesser number of subrogated variables called latent variables. It is also used to remove redundancy in data which may cause multi-co-linearity between independent variables a common problem facing model building like regression analysis. Beside other important aspects like revealing patterns and data screening.

*Following* Table 1*presents the results of Exploratory Factor Analysis (EFA), reliability coefficient* and extracted variance for each construct. Some descriptive measures are also included in Table 1 to show the respondent perception to each construct and the significance of their responses.

**Table 1:** Factor analysis results

Construct	Factor loadings	Reliability coefficient (%)	Variance extracted (%)
*Recognition*		94.4	86.389
My personal well-being is important to my supervisor	0.927		
My supervisor makes me feel that I matter	0.908		
My supervisor is sensitive to my needs	0.947		
I receive congratulations from my supervisor when I reach specific goals	0.935		
*WLB*		94.2	89.625
I often visit my friends and acquaintances	0.953		
Because of my work, I have no free time	0.950		
Because of my work, I neglect my family or friends	0.937		
*HAW*		85.9	78.012
How satisfied are you with the pay you receive for your job?	0.878		
How satisfied are you with the opportunities which exist in this organization for advancement [promotion]?	0.887		
I would be very happy to spend the rest of my career with this organization	0.885		
*Organizational Learning Capability (OLC)*		93.3	83.455
People here often venture into unknown territory	0.795		
It is part of the work of all staff to collect, bring back, and report information about what is going on outside the company	0.836		
There are systems and procedures for receiving, collating and sharing information from outside the company	0.850		
People are encouraged to interact with the environment: competitors, customers, technological institutes, universities, suppliers, etc.,	0.795		
Overall		92.7	85.049

Table 1 illustrates the most reliable and valid instruments to measure each construct. The overall reliability is 92.7% which reveals an excellent internal consistency of measuring the research constructs. It is also true that the minimum reliability is 85.9%. The validity which is a measure of accuracy can be measured by the extracted variance. The overall extracted variance is 85.049%, which affirms strong accuracy among the constructs, compared to 70% as a common practice, see [46], for details. Having obtained to most valid and reliable instruments to measure each construct, we express each construct as a linear combination of its indicators weighted by the corresponding factor loadings to give descriptive statistics to each construct, and hence, test if the average response of each construct is significant using the *t*-test. Following Table 2 presents the results regarding the average of each construct, its standard error, the *t*-value, and the *p* value.

**Table 2:** Statistical summary measures of research constructs

Construct	Sample size	The average	Standard deviation	The standard error	*t*-value	*p* value
Recognition	207	3.80	1.036	0.073	11.039	.000*
WLB	207	2.99	1.055	0.074	− .087-	.931*
HAW	207	3.59	1.002	0.070	8.543	.000*
OLC	207	3.58	0.986	0.069	8.447	.000*

*P ≤ 0.05

As indicated in Table 2, respondents are very positive regarding the internal customer orientation with an average rating 3.8 on 5-likert scale. The *t*-test of whether respondents are neutral in their perception the mean rating = 3 (neutral) reveals significant positive attitude toward the recognition (*p* value = 0.000). In the contrary, Respondents are neutral regarding the work–life balance with an average rating = 2.99 on 5-point Likert scale, and *p* value = 0.931. Respondents have strong positive and significant perception to job happiness with an average rating 3.59 on 5-likert scale and *p* value = 0.000. Likewise, respondents have strong positive and significant perception to the organizational learning with an average rating = 3.58 on 5-liket scale and *p* value = 0.000.

the symbole [*] stands for P ≤ 0.05

### The effect of respondents different demographical characteristics on the research constructs

In this section, we want to discuss the question of how different levels of demographical characteristics perceive the research constructs. Although the two levels of gender (Male, Female) have positive perception to the research constructs, Recognition, WLB, HAW and OLC, the results of the *t*-test confirm no significant differences between the two levels. The results for other Demographical characteristics are the same for the one-way analysis of variance test, among the experience’s level, educational levels. Except for the happiness, age groups have no significant differences between groups levels for the other research constructs. Regarding the HAW construct, age groups less than 50 are less perceptive the issue of HAW compared to those of older age although all groups have positive perception to the issue of HAW. In the following section, we introduce the proposed causal model along with the data fitting.

### Research conceptual model

**Fig. 1:**
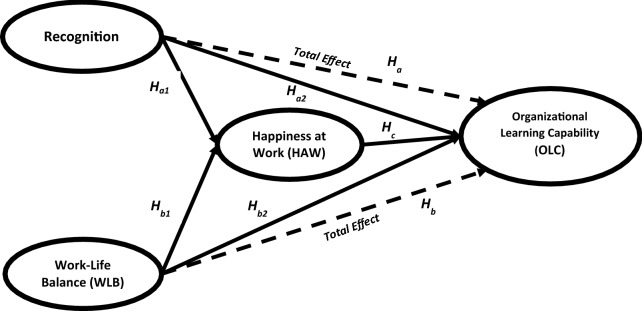
Research conceptual model

As depicted in Fig. 1, both of Recognition and WLB are exogenous variables that stimulate the causal effects process. The effect is transmitted to the mediator construct, HAW directly, which in turns transmits to the outcome construct, OLC directly. At this point, we propose to test the following set of research hypotheses:

#### Research hypotheses

##### H_a_

Recognition has significant positive Total Effect on OLC.

##### H_a1_

Recognition has significant direct effect on HAW.

##### H_a2_

Recognition has significant positive direct effect on OLC.

##### H_a3_

Recognition has significant positive indirect effect on OLC via HAW.

##### H_b_

WLB has significant positive Total Effect on OLC.

##### H_b1_

WLB has significant direct effect on HAW.

##### H_b2_

WLB has significant positive direct effect on OLC.

##### H_b3_

WLB has significant positive indirect effect on OLC via HAW.

##### H_c_

HAW has significant direct Effect on OLC.

To justify the above set of research hypotheses, we utilized Lisrel Software Ver 5.88, to fit the data the proposed structural equation model against several alternatives.

#### The fitted model

The proposed model is fitted against several alternatives to attain several goodness of fit measurements including Normed Fit Index (NFI) = 0.94, Non-Normed Fit Index (NNFI) = 0.94, Parsimony Normed Fit Index (PNFI) = 0.75, Comparative Fit Index (CFI) = 0.96, Incremental Fit Index (IFI) = 0.96, Relative Fit Index (RFI) = 0.93, Critical N (CN) = 65.04, Root Mean Square Residual (RMR) = 0.038, Standardized RMR = 0.038 (Common practice < 0.05), Goodness of Fit Index (GFI) = 0.82(common practice ≥ 0.85), Adjusted Goodness of Fit Index (AGFI) = 0.80 (See Hair et. al. (2015).

#### The correlation structure

Following Table 3 gives the correlation structure between the research constructs.

**Table 3:** Correlation Matrix of ETA and KSI

	HAW	OLC	Rec	WBS
HAW	1.00			
OLC	0.72	1.00		
*p* value	0.000			
Rec	0.58	0.66	1.00	
*p* value	0.000	0.000		
WBS	0.36	0.43	0.37	1.00
*p* value	0.000	0.000	0.000	

As shown in Table 3, there is strong positive correlation between Happiness, and Organization Learning (*r* = 0.72, *p* value = 0.000), Happiness has strong positive and significant correlation with Recognition (*r* = 0.58, *p* value = 0.000), and significant weak positive correlation with Work Balance (*r* = 0.36, *p* value = 0.000). Organization Learning has strong positive and significant correlation with Recognition (*r* = 0.66, *p* value = 0.000) and has significant weak positive correlation with Work Balance (*r* = 0.43, *p* value = 0.000). Recognition has significant weak positive correlation with Work Balance (*r* = 0.37, *p* value = 0.000).

#### The composite reliability cr and average variance extracted AVE

The composite reliability is more efficient measure of internal consistency compare to Cronbach, presented in Table 1. It is calculated after fitting the causal model through the mathematical formula

$${\text{Composite Reliability}} = \frac{{(\sum {\text{Standardized loadings}})^{2} }}{{(\sum {\text{Standardized loadings}})^{2} + \sum \left| {{\text{error}}} \right|}}{ }$$

where standardized loadings and associated errors are calculated by the structure equation technique among LISREL output.

The average variance extracted is a measure accuracy of the model, which can be used as an indicator of construct validity. It is calculated through the following formula

$${\text{Average Variance extracted}} = \frac{{\sum \left( {\text{Standardized loadings}} \right)^{2} }}{{\sum \left( {\text{Standardized loadings}} \right)^{2} + \sum \left| {{\text{error}}} \right|}}$$

As illustrated in Table 4, all composite reliability are above 88.76%, which affirms strong consistence of the instruments to measure constructs [practically above 70% see [46]; likewise, all Average Variance Extracted are above 72.50% which exhibits strong validity of constructs (commonly above 50%, see [46] for details. It is also of interest to check the questionnaire design; in case, it lacks discriminant validity between different constructs.

**Table 4:** The construct reliability and the average variance extracted

Construct	Composite reliability (%)	Average variance extracted (%)	*R*^2^ coefficient of determination
Recognition	96.63	87.78	–
WLB	95.05	86.49	–
HAW	88.76	72.50	36%
OLC	95.53	84.05	63%

#### Evidence of discriminant validity

Discriminant validity is the extent to which latent construct $\eta_{1} {\text{discriminates from the latent construct }} \eta_{2} .$ Farrell [35] argued for a review of discriminant validity assessment in organizational research to establish a high degree of trust and confidence in subsequent results. Although there are several measures to calculate the discriminant validity between a pair of constructs, it is safe to assess discriminant validity or lack of it between each pair of constructs using the formula.

${\text{DV}} = \frac{{r_{{\eta_{1} \eta_{2} }} }}{{\sqrt {{\text{CR}}_{{\eta_{1} }} {\text{CR}}_{{\eta_{2} }} } }}$**,**where $r_{{\eta_{1} \eta_{2} }}$ is the correlation between the two constructs, $\eta_{1}$ and $\eta_{2}$ and ${\text{CR}}_{{\eta_{1} }}$, ${\text{CR}}_{{\eta_{2} }}$ are their construct reliabilities, respectively. Following Table 5 presents value of DV for each pair constructs.

**Table 5:** Discriminant validity between different research constructs

Constructs	HAW	OLC	Recognition	WLB
HAW	1			
OLC	78%	1		
Recognition	62.63%	67%	1	
WLB	39.21%	45.13%	38.61%	1

As shown in Table 5, all measures of DV are less than 78%, which clearly illustrates strong discriminant between each pair of constructs (commonly 85% and above is considered lack of discriminant validity) see [46].

#### Path analysis and the of research hypotheses

Table 6 presents the path analysis results for direct, indirect and total effects along with the verification of the proposed research hypotheses.

**Table 6:** Path analysis results

Effects	Path coefficient	Standard error	*t*-value	*p* value
*Direct effects*				
${\text{Recognition}} \Rightarrow {\text{Happiness}}$	0.52	0.08	6.82	0.000
${\text{Recognition}} \Rightarrow {\text{Org}}.\,{\text{Learning}}$	0.33	0.07	5.09	0.000
${\text{W}}.\,{\text{Balance}} \Rightarrow {\text{Happiness}}$	0.16	0.07	2.40	0.008
${\text{W}}.\,{\text{Balance}} \Rightarrow {\text{Org}}.\,{\text{Learning}}$	0.14	0.05	2.54	0.006
${\text{Happiness}} \Rightarrow {\text{Org}}.\,{\text{Learning}}$	0.48	0.07	6.86	0.000
*Indirect effect*				
${\text{Recognition}} \Rightarrow {\text{Happiness}} \Rightarrow {\text{Org}}.\,{\text{Learning}}$	0.25	0.05	5.14	0.000
${\text{Work}}\,{\text{Balance}} \Rightarrow {\text{Happiness}} \Rightarrow {\text{Org}}.\,{\text{Learning}}$	0.08	0.03	2.30	0.011
*Total effects*				
${\text{Recognition}} \Rightarrow {\text{Org}}.\,{\text{Learning}}$	0.58	0.07	8.73	0.000
${\text{W}}.\,{\text{Balance}} \Rightarrow {\text{Org}}.\,{\text{Learning}}$	0.21	0.06	3.59	0.000

As depicted in Table 6, there is 52% positive and significant effect of Recognition on HAW, which verifies the research hypothesis ${\text{H}}_{{{\text{a}}1}}$, *p* value 0.000. Regarding the partial effect of recognition on OLC, Recognition has 33% significant positive direct effect on OLC, which justifies ${\text{H}}_{{{\text{a}}2}}$, *p* value 0.000. Moreover, Recognition has 25% positive and significant indirect effect on OLC via HAW, *p* valued = 0.000 which partially supports the research hypothesis ${\text{H}}_{{{\text{a}}3}}$. As a conclusion, Recognition has 58% positive and significant total effect on OLC which asserts the main hypothesis ${\text{H}}_{{\text{a}}} ,{ }$ *p* value = 0.000. Therefore, ${\text{H}}_{{\text{a}}}$ is fully supported. On the other hand, WLB has 16% significant positive direct effect on HAW, which confirms the research hypothesis ${\text{H}}_{{{\text{b}}1}}$, *p* value = 0.008. Similarly, WLB has 14% significant positive direct effect on OLC *p* value = 0.006, which affirms the research hypothesis ${\text{H}}_{{{\text{b}}1}}$. In addition, WLB has 8% significant positive indirect effect on OLC, *p* value 0.011, which proves the validity of ${\text{H}}_{{{\text{b}}3}}$. Meanwhile, WLB has 21% significant positive total effect on OLC, *p *value = 0.000 that supports the hypothesis ${\text{H}}_{{{\text{b}}.{ }}}$. As a conclusion, ${\text{H}}_{{{\text{b}}.{ }}}$ is fully supported. Finally, the HAW has 48% significant positive direct effect on OLC, *p* value = 0.000 that supports our hypothesis ${\text{H}}_{{{\text{b}}.{ }}} .$

#### The role of happiness as a mediator

First, let us discuss the direct and indirect effects of Recognition on OLC to identify the real effect of HAW to mediate the relationship between Recognition and OLC. The Direct effect of Recognition on OLC is 33%, while the indirect effect of Recognition on OLC is 25%, and this comparison shows how important the HAW in transmitting the effect. Also, the direct effect of WLB on OLC is 16%, while the indirect effect is 8%. To sum it up, the HAW plays an important role in mediation relationship between exogenous and the outcome variable.

## Discussion and implications

In a nutshell, this research on hands aimed at investigating the impact of specific total rewards components such as Recognition and WLB on achieving an acceptable level of HAW, which meanwhile leads to improving the OLC of an individual organization in the Higher Education (HE) sector in Egypt.

### Direct impact relationships

Regarding the direct impact relationship between recognition and HAW, the initial hypotheses test shows that recognition has a significant positive impact relationship on HAW among academic staff at private HE sectors in Egypt by 52% at confidence level 99.9%, where (*p* < 0.01). Hence, the postulated hypothesis H_a1_ is accepted. With regard to recognition results, multifarious researches had demonstrated a significant positive impact relationship between recognition and HAW among different contexts and samples sizes, and this confirms the current research results concerning H_a1_. For instance, Atan et al. [6] proved such significant positive impact relationship between recognition and HAW among a sample of female employees in various four and five-star hotels in Cyprus. Likewise, Melie et al. [66] conducted their research on a sample of hospitality employees, and they reported the same significant positive impact relationship, as they found that happiness is found in moments of external recognition of professional achievements.

On the other hand, concerning the direct impact relationship between recognition and OLC, it was hypothesized that recognition has a significant positive impact relationship on HAW among academic staff at private HE sectors in Egypt by 33% at confidence level 99.9%, where (*p* < 0.01). Hence, the proposed hypothesis H_a2_ is supported. Accordingly, concerning the recognition findings, many several researches had highlighted clear evidences for a significant positive impact relationship between recognition and OLC among different contexts and sizes of sample, and this supports the current research findings concerning H_a2_. For example, Austin and Harkins [7] in their research on a similar context which is schools have revealed that there is a relationship between recognition and movement toward organizational learning. In parallel to this hype, the work of Nafukho, Graham and Muyia [71] which was applied on 150 workers at carton factories pinpointed the evidence for promoting organizational learning in small-sized business through recognition programs. Similarly, the research conducted by Jain and Moreno [50] conducted in engineering management projects in which data were collected from 205 middle and senior executives. Their work indicated the existence of positive direct impact relationship between employee recognition and organizational learning. Uniformly, Afshari and Hadian [2] have applied their research on a sample of 225 employees from ports and maritime organizations, and they assumed a similar significant impact relationship between recognizing employee talents and OLC.

For the H_b_ the direct impact relationship between WLB and HAW, the proposed hypotheses test demonstrated that WLB has a significant positive impact relationship on HAW among academic staff at private HE sectors in Egypt by 16% at confidence level 99.9%, where (*p* < 0.01). Accordingly, the postulated hypothesis H_b1_ is accepted. After digging deeply in the body of knowledge, it was remarkable that many researches had presented a significant positive impact relationship between WLB and HAW among different contexts and samples sizes, and this supports the current proposed research findings for H_b1_. For example, the findings of the current research are consistent with the results of Badri et al. [9] which presented evidence for a positive direct impact relationship between WLB and HAW who relied in their research on Abu Dhabi QoL Survey conducted previously in 2020.

On the other hand, some other researchers found that there was no significant direct impact relationship between WLB and HAW. For instance, there the work of Dhingra and Dhingra [29], which was mainly applied on 206 doctors in Indian hospitals, revealed in their research that WLB does not necessarily contribute to the achievement of HAW unless for the case of female employees.

In addition, the direct impact relationship between WLB and OLC suggested the existence of a significant positive impact relationship between WLB and OLC among academic staff at private HE sectors in Egypt by 14% at confidence level 99.9%, where (*p* < 0.01). This means that the alleged hypotheses H_b2_ are accepted. The findings of such hypotheses are aligned with the findings of other researchers such as [83]  who conducted their work on sample of 167 medical staff working in allergy units, and they found that organizations can facilitate OLC through achieving some sorts of WLB. Similarly, Charoensukmongkol and Puyod [19] whose research was applied on 522 employees at three public universities in the Philippines, asserted the existence of transformational leadership positively promotes the work–life balance, thus enhancing organizational learning capability.

### Indirect relationships

In this part of the discussion section, the researchers report the indirect impact relationships including the total effect and mediating role of HAW in tunneling the relationship between WLB, recognition and OLC.

Consequently, the findings of H_a_ have suggested that Recognition has 25% positive and significant indirect effect on OLC via HAW, (*p* < 0.01) which partially supports the research hypothesis ${\text{H}}_{{{\text{a}}3}}$. As a conclusion, Recognition has 58% positive and significant total effect on OLC which asserts the main hypothesis ${\text{H}}_{{\text{a}}} ,{ }$(*p* < 0.01). Therefore, ${\text{H}}_{{\text{a}}}$ is fully supported. This means that recognition has significant positive total effect on OLC and partially through the mediating effect of HAW. Consequently, the findings of this research have demonstrated a framework which reveals that employees at the Egyptian private higher education sector who feel highly recognized on their efforts at their universities are more likely to feel happy with their working environments. This proposed framework is mainly consistent with the Social Exchange Theory (SET) developed by Blau in 1964. The SET entails that employees tend to be willing to contribute to their organizations in terms of knowledge and skills, only if they feel that their employers are concerned for their welfare or when their organizations grant them adequate and reasonable job resources [43].

Regarding the findings of H_b_, it was concluded that WLB also has a significant positive total effect on OLC and partially through the mediating effect of HAW. Accordingly, these findings were mainly consistent with the findings of [38, 82, 83, 91, 97] who have asserted that many organizations who have tried to facilitate and enhance their OLC are granting their employees the opportunity to achieve some sorts or balance between their work and personal lives. This means that those employees, especially the academic staff working in Egyptian private universities, should be granted with the sense of WLB in order to be more active and innovative which in turn reinforces the OLC of such universities.

Concerning the findings of H_c_, it was concluded that HAW has a significant positive total effect on OLC and partially mediates the Recognition-OLC and WLB-OLC links. Therefore, these findings were mainly consistent with the findings of Salas-Vallina [82] who have asserted that creating a learning organization is a function of a series of actions that have a significant impact in creating OLC such as: continuous learning opportunities, promoting dialogue, collaboration, shared learning concepts, shared vision, employees’ empowerment which could be all considered as ingredients of HAW and totally directing the organization toward creating OLC.

Generally, this research bridges the gap in the body of knowledge in knowledge management and total rewards management. Therefore, the following theoretical, practical and policy making implications have been revealed:

### Theoretical implications

In fact, the current research is a significant contributor to the field of knowledge management (KM), total rewards management and Happiness body of knowledge. Concerning the KM domain, the present research contributes in terms of defining these total rewards drivers or antecedents that mainly stimulate the OLC. Basically, factors such as recognition and WLB are directly and indirectly affecting OLC. Concerning the HAW construct work and findings, the study contributes to the literature by complementing the theory of Social Exchange (SET). The study advances knowledge of Social Exchange Theory by examining how employees tend to be willing to contribute to their organizations in terms of knowledge and skills only if they feel that their employers are concerned for their welfare. Therefore, HAW is essential to enhance employees’ welfare by providing acceptable satisfaction and positive affection at workplaces. This can include both tangible rewards such as salary and benefits, as well as intangible rewards such as recognition, opportunities for growth and development, and a positive work culture. Factors that can influence an employee's sense of happiness at work include the balance between the rewards and costs of their job, the fit between their personal values and the values of the organization, and the quality of their relationships with supervisors. Additionally, the HAW construct work and findings, it contributes to the existing literature of HRM through demonstrating the mediating role of HAW in tunneling the relationship between recognition, WLB and OLC. Accordingly, a significant theoretical contribution of this research is examining the OLC mechanism through the mediating role of HAW. Finally, the study contributes to the field of knowledge management within an organization. Happy employees may be more likely to share their knowledge and expertise with their colleagues, which can help to build a culture of knowledge sharing and collaboration. Happiness at work can also lead to better teamwork and communication, which can facilitate the sharing and transferring of knowledge within the organization. Thus, happy employees may be more open to new ideas and more likely to take risks, which can lead to increased innovation and learning. Moreover, this research contributes to the building blocks of the SET.

### Practical implications

On the practical side, this research has major implications for both HR professionals and the top management of Egyptian private universities. For HR professionals, organizational learning capability can be facilitated by firstly; developing effective employee recognition programs (monetary and non-monetary) as a crucial element of the total reward strategy which effectively aids in enhancing the university learning capabilities. Moreover, the HR department should highpoint the vital role of the supervisor in implementing the recognition programs. Thus, HR should provide training programs to the supervisors to train them how effectively recognize their subordinates. Secondly, the HR department should design an attractive total reward strategy for the university staff, which contains work–life balance initiatives from family-friendly policies, flexible schedules, 4 days workweek, work from home, and compressed workweek. Not only these initiatives will enhance university learning capabilities, but also it will be a source of competitive advantage that aid in talent attraction and retention. Happy employees may be more motivated, engaged, and productive, which can facilitate a culture of continuous learning and improvement. Happiness at work can also lead to better teamwork and communication and a more positive work environment, all of which can contribute to better learning outcomes. Additionally, happy employees may be more open to new ideas and more likely to take risks, which can lead to increased innovation and learning. Overall, organizations need to create a positive work environment and promote the well-being of their employees to support organizational learning and development. For top management, it is advisable to establish a happiness management department under the supervision of HR to enhance staff satisfaction, recognition, and capabilities. The top management has to work with the HR department to create a positive work environment and promote the well-being of their employees to support effective knowledge management practices. Consequently, continuous learning capabilities, knowledge transfer, collaboration, a shared learning atmosphere, and employee’s empowerment will be achieved.

## Limitations and further researches

The current study has some limitations. First, the application area was limited to private universities in Egypt. Hence, future studies should inspect the study relationships in public universities to provide a holistic view. Furthermore, the research results highlight that happiness at the workplace has a partial mediating effect between recognition and organizational learning capabilities as well as work–life balance and organizational learning capabilities. Accordingly, it is advised to study these relationships in another context. Second, the study relies on a cross-sectional approach, so a longitudinal study may be employed to provide insights regarding the evolution of organizational learning capabilities over a prolonged period. Thirdly, comparing public universities and private can provide an overview of the entire higher education section. Qualitative researches and case studies may provide in-depth insights regarding the research variables. Fourthly, at the mediating level, further research is required to analyze the moderating or mediating effect of other variables, such as organizational culture, knowledge management or organizational forgetting. Additionally, future studies might investigate the relationships between internal customer orientation, job challenge and organizational learning capability.

## Conclusion

In a nutshell, to today’s dynamic work environment, the HR department is exerting tremendous efforts to design innovative total rewards mix such as recognition and work–life balance to improve the organizational learning capabilities of the organization. The results demonstrate that when employees feel highly recognized, particularly by their direct supervisors for their effort, they are more likely to feel happy with their working environments. Numerous empirical researches indicate that employees tend to be willing to contribute to their organizations regarding knowledge and skills when they feel that their employers are concerned for their welfare. This reveals that enhancing organizational learning capabilities depends to a large extent on employee recognition, work–life balance as well as happiness at the workplace. Therefore, HR managers are required to develop an effective rewards mix containing; active employee recognition programs, work–life balance initiatives, and happiness initiatives that stimulate learning capabilities, knowledge transfer, and collaboration.

## Data Availability

The data for our analysis are available from the corresponding authors and could be furnished and submitted upon request.

## Abbreviations

WLB: Work–life balance
HAW: Happiness at work
OLC: Organizational learning capabilities
HR: Human resources
HRM: Human resources management
SET: Social exchange theory
WFC: Work–family conflict
